# The extracellular matrix protein mindin as a novel adjuvant elicits stronger immune responses for rBAG1, rSRS4 and rSRS9 antigens of *Toxoplasma gondii*in BALB/c mice

**DOI:** 10.1186/1471-2334-14-429

**Published:** 2014-08-04

**Authors:** Xiaojing Sun, Mei Mei, Xu Zhang, Fusong Han, Boyin Jia, Xiaoyan Wei, Zhiguang Chang, Huijun Lu, Jigang Yin, Qijun Chen, Ning Jiang

**Affiliations:** Key Laboratory of Zoonosis, College of Veterinary Medicine, Jilin University, Xi An Da Lu 5333, Changchun, 5333 China; Medical Clinic, College of Veterinary Medicine, Jilin University, Xi An Da Lu 5333, Changchun, 5333 China

**Keywords:** Extracellular matrix, Mindin, Adjuvant, Immune response

## Abstract

**Background:**

Vaccines are the most effective agents to control infections. However, recombinant vaccines often do not elicit strong immune responses. Protein antigens combined with proper adjuvants have been widely used to induce immune responses, especially the humoral immune responses, against various pathogens, including parasites. The extracellular matrix protein mindin has been recognised as an immune facilitator for initiating innate immune responses. It has therefore been expected to be a potentially potent adjuvant in the development of novel vaccines. The aim of this study was to investigate whether mindin could facilitate the induction of antigen-specific immune responses to recombinant antigens (rBAG1, rSRS4 and rSRS9) of *Toxoplasma gondii* in BALB/c mice.

**Methods:**

In this study, we explored the adjuvant effect of the recombinant mindin in the generation of specific Th1 and Th2 responses to each of three *T. gondii* antigens, BAG1, SRS4 and SRS9. All mice in the experimental groups received either antigen alone or in combination with Freund’s adjuvant or with the recombinant mindin. The immune responses after immunisation were measured by ELISA and lymphoproliferative assays. The immunised mice were challenged with live *T. gondii* tachyzoites, and the protection efficiency was compared between the groups.

**Results:**

Our results revealed that mindin as an adjuvant could facilitate the recombinant proteins to efficiently stimulate humoral and cellular responses, including antigen-specific IgG1 and IgG2a, as well as lymphocyte proliferation. Furthermore, significantly improved protection against *T. gondii* infection was observed in the mindin group compared with that of Freund’s adjuvant and no-adjuvant groups.

**Conclusions:**

The extracellular matrix protein mindin can effectively induce antigen-specific humoral and cell-mediated immune responses. Our study provides a valuable basis for the development of an efficient, safe, non-toxic vaccine adjuvant for future use in humans and animals.

**Electronic supplementary material:**

The online version of this article (doi:10.1186/1471-2334-14-429) contains supplementary material, which is available to authorized users.

## Background

Vaccine adjuvants facilitate the production of long-lasting, efficient and specific immune responses and improve the protective effect of vaccines due to a higher antibody yield and the persistence of antibodies, as well as functional T cells at high levels. Currently, the most common adjuvant used in experimental animals is Freund’s adjuvants, which can enhance strong antigen-specific immune responses. However, it causes strong inflammation and necrosis at the injection site, which prevents its use in vaccine development. Aluminium-derived adjuvants are often used in clinical trials and have the reputation of safety and the facilitation of long-lasting antibody responses [1], but the effect on cell-mediated immunity remains questionable when used along with small immunogenic antigens. To develop safe and effective adjuvants for enhancing both humoral and cellular immune responses, we focused on the selection of novel immunofacilitators based on their roles in initiating innate and adaptive immune responses.

Mindin (also known as spondin 2) belongs to the mindin-F-spondin family of secreted extracellular matrix (ECM) proteins that includes mammalian F-spodin, zebrafish mindin 1 and 2, and Drosophila M-spondin [2–5]. Mindin has been known to essentially initiate innate immunity and serve as a bridge between innate and adaptive immunity [6]. Previous reports indicated that mindin-deficient mice had an impaired capability to clear influenza virus and bacterial infection, and mindin-deficient macrophages exhibit defective responses to a broad spectrum of microbial stimuli. This is because mindin is a pattern recognition molecule and can directly bind to sugar moieties in bacterial cell walls [7] and influenza virus particles to initiate innate immune responses, as well as function as an opsonin for macrophage phagocytosis of bacteria [8]. Interestingly, even recombinant mindin alone is capable of promoting viral clearance in wild-type mice [6]. Possible mechanisms for mindin’s effects on immunity system include binding to a cell surface receptor, activating synergistic signal transduction pathways for proinflammatory cytokine production, interacting with other pattern recognition receptors (PRR) (such as the Toll-like receptors) and enhancing their recognition of bacterial products, inducing bacterial phagocytosis to enhance the immune response to infection, or a combination of these mechanisms [7]. Based on these findings, mindin has been considered as a novel vaccine adjuvant candidate in view of its superior immune-enhancing capabilities.

*Toxoplasma gondii*, a member of the Apicomplexa phylum, is an obligatory intracellular and opportunistic parasite that infects both warm-blooded animals and human beings to cause toxoplasmosis [9]. This parasite is a highly prevalent, zoonotic pathogen of medical, veterinary and economic importance [10, 11]. Acquired toxoplasmosis is usually an asymptomatic disease of adults, which most likely persists for the lifetime of the hosts. However, this disease can be quite severe or even fatal for immunocompromised patients, such as those with AIDS, organ transplant recipients, or those with neoplastic diseases [12, 13], and the sequelae associated with diseases include blinding chorioretinitis, lymphadenopathy, encephalitis and/or death [14]. Toxoplasmosis has long been a disease of worldwide importance for public health and economic development. Consequently, the development of an effective vaccine for controlling the impact of this disease is urgently needed.

Currently, the best developed vaccine is the live, attenuated tachyzoite S48 [15], which has not been widely applied because of its high cost, side effects, and short shelf life. More importantly, attenuated vaccines carry a risk of unexpected harmful reverse mutations and accidental infections of humans [16]. To overcome these issues, most of current research has been focused on searching for subunit or recombinant vaccines. The SAG1-related sequence (SRS) super-family, encoding glycophosphatidylinositol (GPI)-anchored surface proteins [17], are the crucial surface antigens of *T. gondii* that participate in the process of host cell attachment and regulate the virulence of these parasites, which could be a promising vaccine candidate against toxoplasmosis [18]. Furthermore, the SRS9 molecule, a bradyzoite-specific SRS antigen, has been suggested to be an important target of the host immune response in the mouse intestine [19]. SRS4 has been shown to be able to elicit strong antibody responses in humans infected by *T. gondii* and has been considered as a diagnostic and/or vaccine antigen [20, 21]. In addition, BAG1, a *T. gondii* 30-kDa cytosolic heat-shock protein, preferentially expressed at the bradyzoite stage, is very immunogenic because of its induction of early humoral and cell-mediated immune responses upon infection in humans [22–24]. Therefore, we chose BAG1, SRS4 and SRS9 of *T. gondii* as vaccine candidates to assess the immune-enhancing effect of mindin. The function of mindin as a novel adjuvant for the *T. gondii* antigens BAG1, SRS4 and SRS9 was evaluated through an analysis of the induction of antigen-specific antibodies, lymphocyte proliferation and the immune protection capacity in challenge experiments. The results showed that the extracellular matrix protein mindin is a very potent adjuvant molecule owing to its enhancement of both humoral and cell-mediated immune responses.

## Methods

### Animals and parasites

Female BALB/c and male Kunming strain outbred mice aged 8 to 10 weeks were used in all experiments. Kunming mice were used to maintain and passage *T. gondii* tachyzoites, whereas BALB/c mice were used in the immunisation experiments. The permission to work with laboratory animals was obtained from the Ethical Committee of the Institute of Zoonosis, Jilin University, China (Permission number 2008-IZ-20). Tachyzoites of the RH strain of *T. gondii* were harvested from the peritoneal fluid of Kunming mice after intraperitoneal infection.

### Generation of recombinant proteins

The *Mus musculus spon 2* gene-encoding extracellular matrix protein mindin was chemically synthesised with restriction sites (BamH*I* and Hind*III*) at the 5′ and 3′ ends, respectively. The gene fragment was subcloned into the plasmid pET-22b (Qiagen, Düsseldorf, Germany) to construct an expression plasmid, which was subsequently confirmed by enzymatic digestion and sequencing. After transformation of the plasmid into BL21 (DE3) competent cells, the expression of the His-tag fusion protein was induced by the addition of IPTG at 37°C for 5 h. The recombinant protein in inclusion bodies was purified by Ni-affinity chromatography (GE Healthsystems, Uppsala, Sweden) according to the manufacturer’s instructions. The inclusion bodies were solubilised by 6 M urea, and the purified, denatured mindin was refolded by dialysis in PBS containing urea (initial concentration was 6 M, then decreased by 1 M/dialysis) and 0.5 M arginine. Subsequently, the refolded protein was dialysed 5 times against the dialysis buffer to remove arginine changing from 0.5 M-0 M buffers, 12 h/time-point. Finally, the protein was confirmed by SDS-PAGE and Western blotting.

The recombinant *T. gondii* antigens (rBAG1, rSRS4 and rSRS9) were generated as described in our previous study [25].

### Immunisation schedule and serum collection

Animals were immunised with one of the three recombinant antigens (rBAG1, rSRS4 and rSRS9) formulated with complete Freund’s adjuvant (CFA)/incomplete Freund’s adjuvant (IFA) (Sigma–Aldrich, St. Louis, MO, USA), the recombinant protein mindin or PBS. To formulate the Freund’s adjuvant emulsion, the antigens were mixed with an equal volume of adjuvant solution in two syringes connected with an adaptor (Sigma). Each test group contained eleven mice and the control groups with the same number of animals receiving the CFA/IFA, mindin or PBS alone, respectively.

All mice were immunised intramuscularly with the antigen-adjuvant mixtures on weeks 2, 4, 6 and 8. The amount of each recombinant antigen used for each immunisation was 20 μg/mouse. Serum samples were collected from all animals in each group prior to the first immunisation and 10 days after each immunisation. Sera were separated from blood by centrifugation at 2000 rpm for 15 min and stored at −80°C until further analysis.

### Detection of specific antibody responses by ELISA

IgG levels in the collected sera were determined by an enzyme-linked immunosorbent assay (ELISA). Maxisor micro-ELISA plates (Nalge Nunc International, IL, USA) were coated with 50 μl per well of the *T. gondii* recombinant antigens (rBAG1, rSRS4 and rSRS9, respectively) in a concentration of 5 μg/ml at 4°C overnight. The plates were washed four times with PBS containing 0.05% Tween 20 and blocked for 1 h at 37°C with 100 μl per well of 3% bovine serum albumin (BSA) in coating buffer (15 mM Na_2_CO_3_, 35 mM NaHCO_3_, pH 9.6). The sera of immunised mice diluted from 1:1000 to 1:128,000 were added in triplicate and allowed to incubate for 1 h at 37°C. Plates were washed, and bound IgG was detected by incubation for 1 h with alkaline phosphatase-conjugated goat anti-mouse IgG (1:20,000, Sigma). Finally, 50 μl of pNPP [4-Nitrophenyl phosphate disodium salt hexahydrate] (Sigma, St. Louis, USA) and 9.7% diethanolamine (pH 9.8) were used to detect antigen-antibody reactions. The optical density (OD) was read at 405 nm in a Biotek micro-ELISA auto-reader 808 (Bio-TEK Instruments, Winooski, USA).

For typing the IgG subclasses, the three fusion proteins were each coated on ELISA plates as described previously and incubated with sera (1:1000) from immunised mice. Antigen-specific IgG subclasses were thereafter identified with commercial kits (BioLegend San Diego, CA, USA) according to the protocols provided by the manufacturer.

### Lymphocyte proliferation assays

Three mice from each group were euthanised two weeks after the last immunisation. Their spleens were removed under sterile conditions. Single-cell suspensions were obtained by filtration through a 200-mesh copper grid. After separation with mouse Percoll (Sigma) used according to the manufacturer’s instructions, the remaining splenocyte suspensions were diluted to a final concentration of 5 × 10^6^ cells/ml in RPMI 1640 with 10% foetal bovine serum (Sigma). Splenocyte suspensions (100 μl/well) were plated into 96-well cell culture plates. The cells were stimulated with 20 μg/ml rBAG1, rSRS4 and rSRS9 at 37°C for 60–72 h in the presence of 5% CO2 followed by the addition of 10 μl/well MTT solutions (5 mg/ml). The plates were then incubated for 4 h at 37°C followed by an addition of 150 μl/well DMSO solution. The sample absorbance at 570 nm was then measured. Concanavalin A (10 μg/ml, Sigma) was used as a positive control, and cells cultured with media alone were used as negative controls. The growth index was calculated according to the formula, Growth Index (GI) = (OD_test_-OD_negative_)/(OD_positive_)-OD_negative_).

### Challenge infection

Eight mice in each group were challenged intraperitoneally with 250 tachyzoites of *T. gondii* RH strain/mouse 2 weeks after the last immunisation. The mice were carefully observed for 20 days.

### Statistical analysis

GraphPad Prism 5 (GraphPad InStatt Software, San Diego, California) and one-factor analysis of variance (ANOVA) were used in this study. Data are expressed as the mean ± standard deviation (SD). The differences between groups were considered to be significant when the P value was less than 0.05. The Kaplan-Meier survival curves were generated with the MedCalc statistical software (http://www.medcalc.org/manual/kaplan-meier.php).

## Results and discussion

### The expression and purification of the recombinant extracellular matrix protein mindin

**Figure 1:**
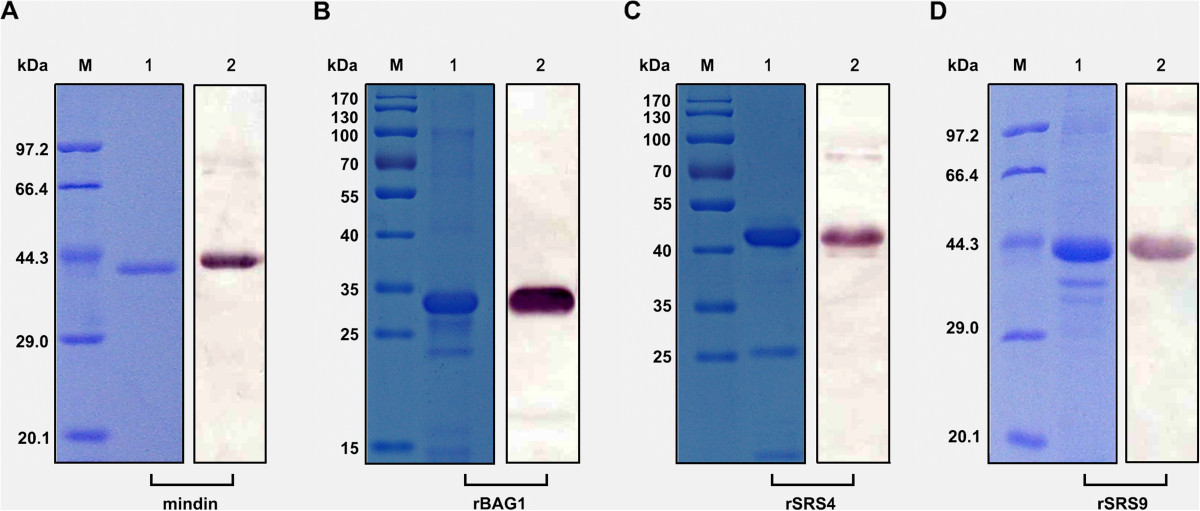
**Purified and confirmation of recombinant proteins.** His-tagged mindin, rBAG1, rSRS4 and rSRS9 fusion proteins were purified, and the quality of the recombinant proteins was determined by a Coomassie-stained SDS-PAGE gel (lane 1 in **A**, **B**, **C** and **D**) and Western blot (lane 2 in **A**, **B**, **C** and **D**). M lane is the protein marker.

The recombinant mindin and rBAG1, rSRS4 and rSRS9 were purified by Ni-affinity chromatography and analysed by SDS-PAGE and Western blotting. The recombinant proteins appeared predominantly as single bands (Figure 1), which were sufficiently pure for the subsequent experiment.

### Humoral immune responses are induced by vaccination with antigens combined with different adjuvants

**Figure 2:**
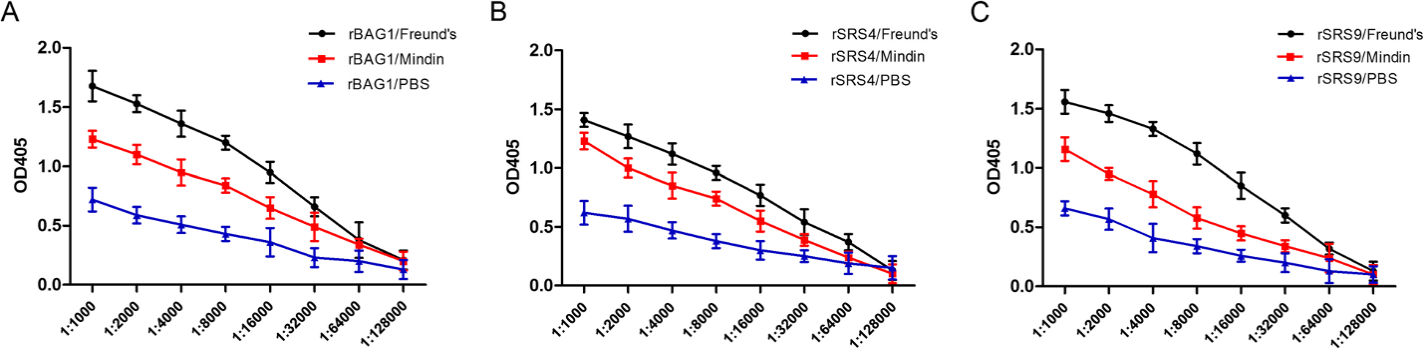
**Antigen-specific IgG responses in different immunised mice after the final immunisation.** BALB/c mice were immunised with rBAG1 **(A)**, rSRS4 **(B)**, **or** rSRS9 **(C)** in combination with Freund’s adjuvant (black) or mindin adjuvant (red) or **as** proteins alone (blue). The IgG response was detected on week 9 (2 weeks after final immunisation). IgG levels are presented as optical density (OD) values at 405 nm with sera diluted from 1:1000 to 1:128,000. The statistical differences between the Freunds’ and the Mindin groups were significant in groups immunised with rBAG1 and rSRS9 **(A and C)**, but not in the groups immunised with rSRS4 **(C)**.

In order to evaluate both the immunogenicity and adjuvant effect of recombinant mindin, BALB/c mice were immunised with rBAG1, rSRS4 and rSRS9 in combination with different adjuvants (Freund’s adjuvant and mindin) or recombinant antigens alone, and antigen-specific IgG antibodies were measured by ELISA. The vaccination of mice with recombinant antigens formulated with Freund’s adjuvant or the mindin adjuvant induced significantly higher levels of IgG than found with antigens alone (p < 0.05; Figure 2). Mindin, therefore, had an adjuvant potency and could significantly increase the antigenicity of the *T. gondii* proteins.

**Figure 3:**
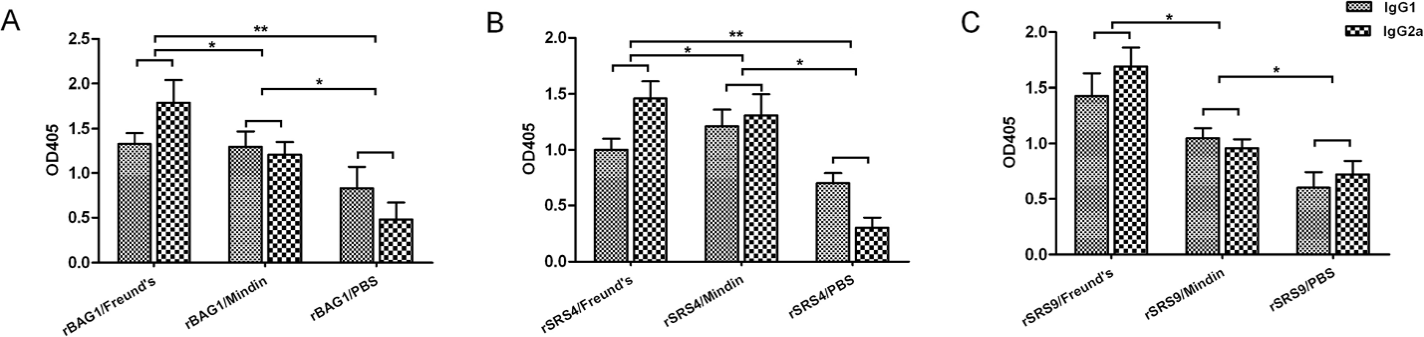
**IgG subclasses in the sera of immunised mice.** The IgG subclass responses to recombinant antigens were detected after the last immunisation. The values shown for each group are the mean ± SD of the antibody levels. **(A)**: IgG subclass responses of rBAG1 antigen group; **(B)**: IgG subclass responses of rSRS4 antigen group; **(C)**: IgG subclass responses of rSRS9 antigen group.

Because the antibody subclass is a measurement of the relative contribution of Th2- versus Th1- type immune responses, we further analysed the IgG subtypes to assess the type of the humoral immunity induced by the different antigen-adjuvant combinations. In mice, the production of IgG1 versus IgG2a is widely interpreted as a reflection of Th2- or Th1 reactivity [26, 27]. In this study, ratios of IgG1/IgG2a levels in sera collected from immunised mice were calculated after the final immunisation. We determined that the levels of IgG1 and IgG2a from BALB/c mice immunised with antigens in combination with Freund’s adjuvant or the mindin adjuvant were clearly higher than those from mice immunised with recombinant antigens alone after the last immunisation (Figure 3). BALB/c mice immunised with the antigens in combination with mindin generated a mixed Th1/Th2 response with similar levels of IgG1 and IgG2a, whereas mice immunised with Freund’s adjuvant generated a pro-Th1-type response, as revealed by a lower IgG1/IgG2a ratio. Immunisation with protein alone induced predominantly IgG1 in BALB/c mice, suggesting a Th2-biased immune response. Thus, it appears that mindin could facilitate the generation of relatively balanced responses by the hosts. This result may be more significant for protection against acute toxoplasmosis. During the initial phase of *T. gondii* infection, the parasites proliferate quickly without host immune pressure. With the development of host immune responses, the parasites can be inhibited by both Th1 and Th2 responses. It is well known that antigen-specific IgGs can block the *T. gondii* techyzoite invasion of host cells and intracellular development [28–30], whereas TNF-α and IFN-γ are important factors in the suppression of parasite proliferation, especially by constraining the dormant form of bradyzoites [31, 32]. Thus, balanced responses would be able to produce optimal immunoprotection [33].

### The cellular immune responses of a splenocyte proliferation assay *in vitro*

**Figure 4:**
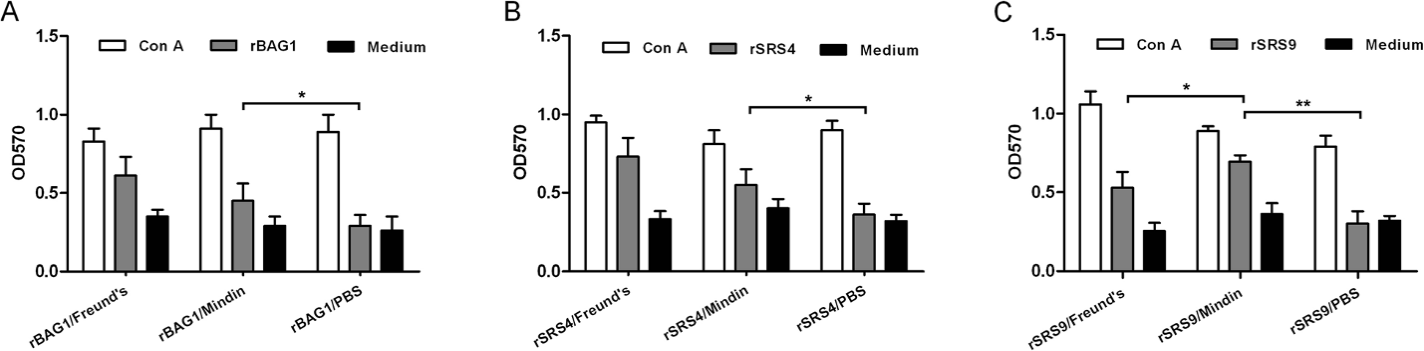
**Lymphocyte proliferation responses of immunised mice.** All groups were intramuscularly immunised four times, and after priming, antigen-stimulated spleen lymphocytes were prepared from three mice per group 2 weeks after the last immunisation, and their subsequent proliferation responses were analysed and expressed as stimulation index (SI) values. Results are presented as the mean of SI values ± SD. **(A)**: Stimulation of rBAG1 antigen; **(B)**: Stimulation of rSRS4 antigen; **(C)**: Stimulation of rSRS9 antigen. The statistical difference between different groups was considered to be significant when the P value was less than 0.05 (* means P < 0.05, ** means P < 0.01 and *** means P <0.001).

The splenocytes from immunised mice of different groups were prepared 2 weeks after the last immunisation to assess splenocyte proliferation *in vitro* (Figure 4). After priming, marked specific lymphoproliferation was observed in splenocyte cultures from mice immunised with recombinant antigens combined with Freund’s adjuvant and mindin adjuvant when stimulated with rBAG1, rSRS4 or rSRS9 (p < 0.05) (Figure 4). This proliferation was further increased when stimulated with Concanavalin A as a positive control. In contrast, splenocytes from mice immunised with antigens alone showed very weak responses when stimulated with any of the three recombinant antigens. A successful vaccine should trigger both humoral and cellular immune responses after immunisation. Our data suggested that splenocytes from mice immunised with rSRS9 formulated with mindin proliferated more efficiently than those from mice immunised with Freund’s adjuvants. Though immunisation with rBAG1 and rSRS4 elicited significantly higher Th1 responses than the medium control group, but the responses were not as significant as that of the group immunised with the rSRS9 antigen (Figure 4). The data further supported the previous report that immunisation with SRS antigen can elicit an antigen-specific Th1 response marked by elevated IFN-γ production [34]. Thus, rSRS9 along with the mindin adjuvant may be an ideal vaccine combination and may induce both humoral and cellular immune responses simultaneously with high efficiency.

### *T. gondii*antigens combined with mindin elicited protective immunity against a lethal challenge in BALB/c mice

**Figure 5:**
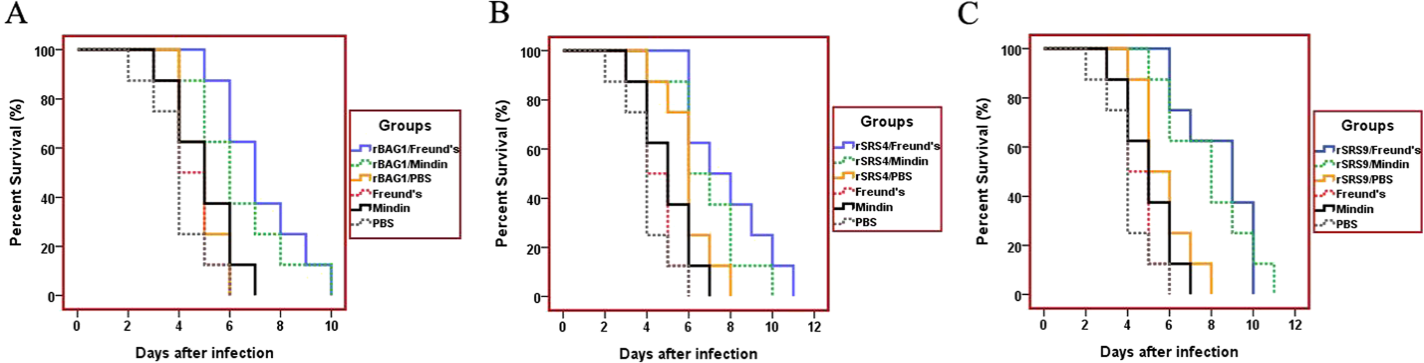
**Survival of immunised mice after a lethal tachyzoite challenge.** Groups of mice were intraperitoneally immunised with the recombinant antigens in combination with the Freund’s adjuvant (light blue), mindin adjuvant (green), PBS (yellow), or Freund’s adjuvant alone (pink), mindin adjuvant alone (black), and PBS alone (grey). Nine weeks after the immunisation, all experimental mice were intraperitoneally infected with 250 live tachyzoites of *T. gondii* (RH strain). Animals were observed daily for 20 days, and the final survival rates were recorded. **(A)**: Mouse group immunised with rBAG1 antigen and controls; **(B)**: Mouse group immunised with rSRS4 antigen and controls; **(C)**: Mouse group immunised with rSRS9 antigen and controls. Mice immunised with the recombinant proteins in combination with either Freund’s adjuvant or mindin showed most prolonged survival time. The Kaplan-Meier survival curves were generated with the MedCalc statistical software (http://www.medcalc.org/manual/kaplan-meier.php).

To evaluate the immuno-protection effect to *T. gondii* infection induced by immunisation of the antigens combined with different *T. gondii* antigens, the immunised mice were challenged intraperitoneally (i.p.) with 250 tachyzoites of *T. gondii* RH strain 2 weeks after the last immunisation, and mortality was monitored daily. The survival rate of the various groups is shown in Figure 5. The data revealed that effective and significant protection was obtained in the mice immunised with recombinant antigens combined with Freund’s adjuvant and mindin adjuvant, respectively. Mice immunised with either antigen alone or adjuvant alone did not show any resistance to the challenge, and all died within 6 days after challenge (P > 0.05). The reason that immunisation with the antigens did not provide immunity against lethal challenge may be due to the less expression of the antigens in the parasites. The two antigens, BAG1 and SRS9 were predominantly expressed at the bradyzoite stage of the parasite and the immunised mice were challenged with tachyzoites. These results, however, demonstrated that mindin as an adjuvant can elicit similar immune-stimulating effects as that of Freund’s adjuvant. Considering the fact that fewer, if any, side effects were produced, mindin could be a promising vaccine adjuvant worthy of further investigation.

## Conclusions

This study has proven that the extracellular matrix protein mindin acts as a potent vaccine adjuvant, which could enhance both humoral and cellular immune responses against *Toxoplasma* infection. The precise mechanism by which mindin promotes immune responses, however, remains open to further investigation.

## Authors’ original submitted files for images

Below are the links to the authors’ original submitted files for images. Authors’ original file for figure 1 Authors’ original file for figure 2 Authors’ original file for figure 3 Authors’ original file for figure 4 Authors’ original file for figure 5

## References

[1] Slingluff CL, Yamshchikov G, Neese P, Galavotti H, Eastham S, Engelhard VH, Kittlesen D, Deacon D, Hibbitts S, Grosh WW, Petroni G, Cohen R, Wiernasz C, Patterson JW, Conway BP, Ross WG (2001). Phase I trial of a melanoma vaccine with gp100(280–288) peptide and tetanus helper peptide in adjuvant: immunologic and clinical outcomes. Clin Cancer Res.

[2] Klar A, Baldassare M, Jessell TM (1992). F-spondin: a gene expressed at high levels in the floor plate encodes a secreted protein that promotes neural cell adhesion and neurite extension. Cell.

[3] Higashijima S, Nose A, Eguchi G, Hotta Y, Okamoto H (1997). Mindin/F-spondin family: novel ECM proteins expressed in the zebrafish embryonic axis. Dev Biol.

[4] Umemiya T, Takeichi M, Nose A (1997). M-spondin, a novel ECM protein highly homologous to vertebrate F-spondin, is localized at the muscle attachment sites in the Drosophila embryo. Dev Biol.

[5] Feinstein Y, Borrell V, Garcia C, Burstyn-Cohen T, Tzarfaty V, Frumkin A, Nose A, Okamoto H, Higashijima S, Soriano E, Klar A (1999). F-spondin and mindin: two structurally and functionally related genes expressed in the hippocampus that promote outgrowth of embryonic hippocampal neurons. Development.

[6] Jia W, Li H, He YW (2008). Pattern recognition molecule mindin promotes intranasal clearance of influenza viruses. J Immunol.

[7] McDonald C, Nunez G (2004). Mindin the fort. Nat Immunol.

[8] He YW, Li H, Zhang J, Hsu CL, Lin E, Zhang N, Guo J, Forbush KA, Bevan MJ (2004). The extracellular matrix protein mindin is a pattern-recognition molecule for microbial pathogens. Nat Immunol.

[9] Tenter AM, Heckeroth AR, Weiss LM (2000). *Toxoplasma gondii*: from animals to humans. Int J Parasitol.

[10] Cenci-Goga BT, Rossitto PV, Sechi P, McCrindle CM, Cullor JS (2010). *Toxoplasma* in animals, food, and humans: an old parasite of new concern. Foodborne Pathog Dis.

[11] Innes EA, Bartley PM, Buxton D, Katzer F (2009). Ovine toxoplasmosis. Parasitology.

[12] Montoya JG, Liesenfeld O (2004). Toxoplasmosis. Lancet.

[13] Meng M, He S, Zhao G, Bai Y, Zhou H, Cong H, Lu G, Zhao Q, Zhu XQ (2012). **Evaluation of protective immune responses induced by DNA vaccines encoding*****Toxoplasma gondii*****surface antigen 1 (SAG1) and 14-3-3 protein in BALB/c mice**. Parasit Vectors.

[14] Havelaar AH, Kemmeren JM, Kortbeek LM (2007). Disease burden of congenital toxoplasmosis. Clin Infect Dis.

[15] Buxton D, Thomson K, Maley S, Wright S, Bos HJ (1991). Vaccination of sheep with a live incomplete strain (S48) of *Toxoplasma gondii* and their immunity to challenge when pregnant. Vet Rec.

[16] Ismael AB, Sekkai D, Collin C, Bout D, Mevelec MN (2003). The MIC3 gene of *Toxoplasma gondii* is a novel potent vaccine candidate against toxoplasmosis. Infect Immun.

[17] Tomavo S (1996). The major surface proteins of *Toxoplasma gondii*: structures and functions. Curr Top Microbiol Immunol.

[18] He XL, Grigg ME, Boothroyd JC, Garcia KC (2002). Structure of the immunodominant surface antigen from the *Toxoplasma gondii* SRS superfamily. Nat Struct Biol.

[19] Kim SK, Boothroyd JC (2005). Stage-specific expression of surface antigens by *Toxoplasma gondii* as a mechanism to facilitate parasite persistence. J Immunol.

[20] Lunden A, Parmley SF, Bengtsson KL, Araujo FG (1997). Use of a recombinant antigen, SAG2, expressed as a glutathione-S-transferase fusion protein to immunize mice against *Toxoplasma gondii*. Parasitol Res.

[21] Howe DK, Crawford AC, Lindsay D, Sibley LD (1998). The p29 and p35 immunodominant antigens of Neospora caninum tachyzoites are homologous to the family of surface antigens of *Toxoplasma gondii*. Infect Immun.

[22] Bohne W, Gross U, Ferguson DJ, Heesemann J (1995). Cloning and characterization of a bradyzoite-specifically expressed gene (hsp30/bag1) of *Toxoplasma gondii*, related to genes encoding small heat-shock proteins of plants. Mol Microbiol.

[23] Parmley SF, Weiss LM, Yang S (1995). Cloning of a bradyzoite-specific gene of *Toxoplasma gondi*i encoding a cytoplasmic antigen. Mol Biochem Parasitol.

[24] Di Cristina M, Del Porto P, Buffolano W, Beghetto E, Spadoni A, Guglietta S, Piccolella E, Felici F, Gargano N (2004). The *Toxoplasma gondii* bradyzoite antigens BAG1 and MAG1 induce early humoral and cell-mediated immune responses upon human infection. Microbes Infect.

[25] Sun X, Lu H, Jia B, Chang Z, Peng S, Yin J, Chen Q, Jiang N (2013). A comparative study of *Toxoplasma gondii* seroprevalence in three healthy Chinese populations detected using native and recombinant antigens. Parasit Vectors.

[26] Du C, Nilsson S, Lu H, Yin J, Jiang N, Wahlgren M, Chen Q (2010). Immunogenicity of the *Plasmodium falciparum* Pf332-DBL domain in combination with different adjuvants. Vaccine.

[27] Chuang SC, Ko JC, Chen CP, Du JT, Yang CD (2013). Induction of long-lasting protective immunity against *Toxoplasma gondii* in BALB/c mice by recombinant surface antigen 1 protein encapsulated in poly (lactide-co-glycolide) microparticles. Parasit Vectors.

[28] Hauser WE, Remington JS (1981). Effect of monoclonal antibodies on phagocytosis and killing of *Toxoplasma gondii* by normal macrophages. Infect Immun.

[29] Johnson AM, McDonald PJ, Neoh SH (1983). Monoclonal antibodies to *Toxoplasma* cell membrane surface antigens protect mice from toxoplasmosis. J Protozool.

[30] Mineo JR, Khan IA, Kasper LH (1994). *Toxoplasma gondii*: a monoclonal antibody that inhibits intracellular replication. Exp Parasitol.

[31] Yap GS, Sher A (1999). Cell-mediated immunity to *Toxoplasma gondii*: initiation, regulation and effector function. Immunobiology.

[32] Stutz A, Kessler H, Kaschel ME, Meissner M, Dalpke AH (2012). Cell invasion and strain dependent induction of suppressor of cytokine signaling-1 by *Toxoplasma gondii*. Immunobiology.

[33] Dallman MJ (1995). Cytokines and transplantation: Th1/Th2 regulation of the immune response to solid organ transplants in the adult. Curr Opin Immunol.

[34] Cleary MD, Singh U, Blader IJ, Brewer JL, Boothroyd JC (2002). *Toxoplasma gondii* asexual development: identification of developmentally regulated genes and distinct patterns of gene expression. Eukaryot Cell.

[CR35] The pre-publication history for this paper can be accessed here:http://www.biomedcentral.com/1471-2334/14/429/prepub

