# Supplementary figures and images for: The extracellular matrix protein mindin as a novel adjuvant elicits stronger immune responses for rBAG1, rSRS4 and rSRS9 antigens of Toxoplasma gondiiin BALB/c mice
Source: BMC Infect Dis. 2014 Aug 4;14:429. doi: 10.1186/1471-2334-14-429 (PMC4131031; doi:10.1186/1471-2334-14-429)

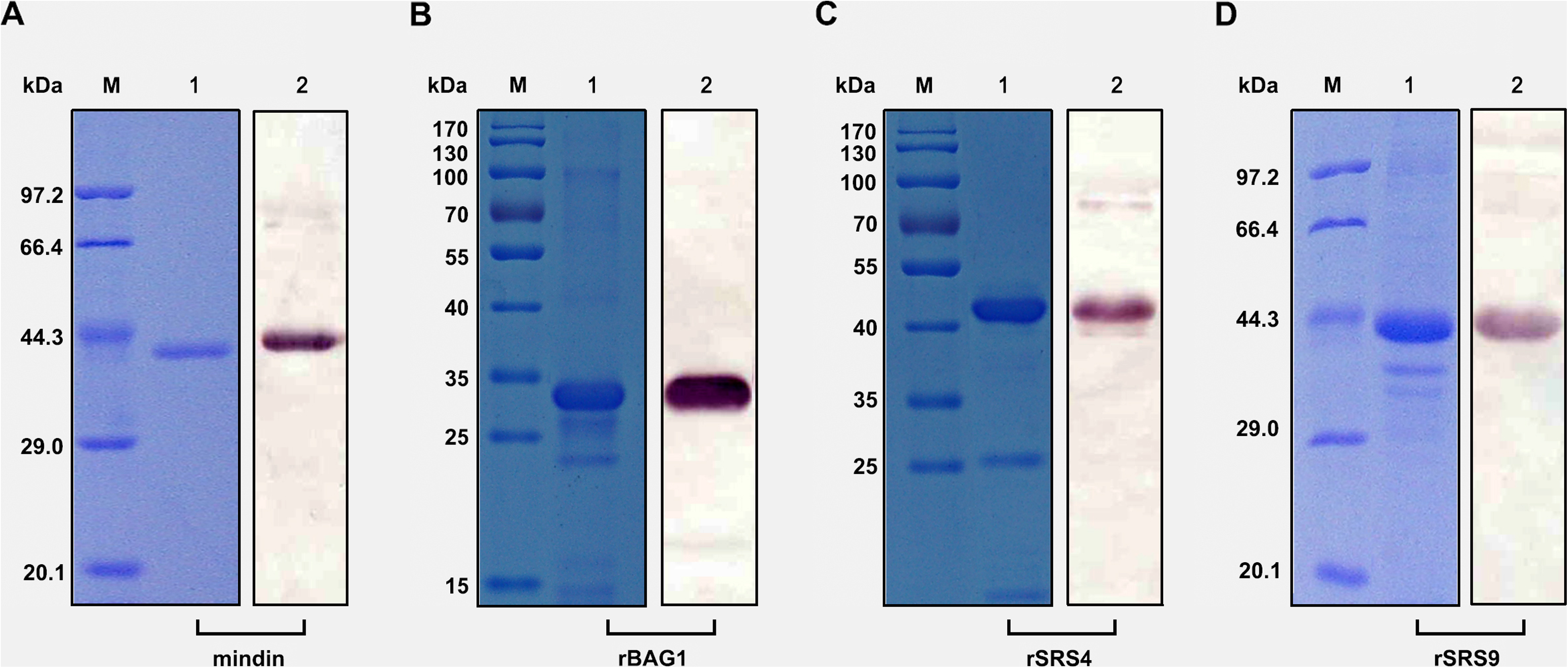

Supplement: Supplementary file 1 — Authors’ original file for figure 1 [file 12879_2014_3729_MOESM1_ESM.tif]

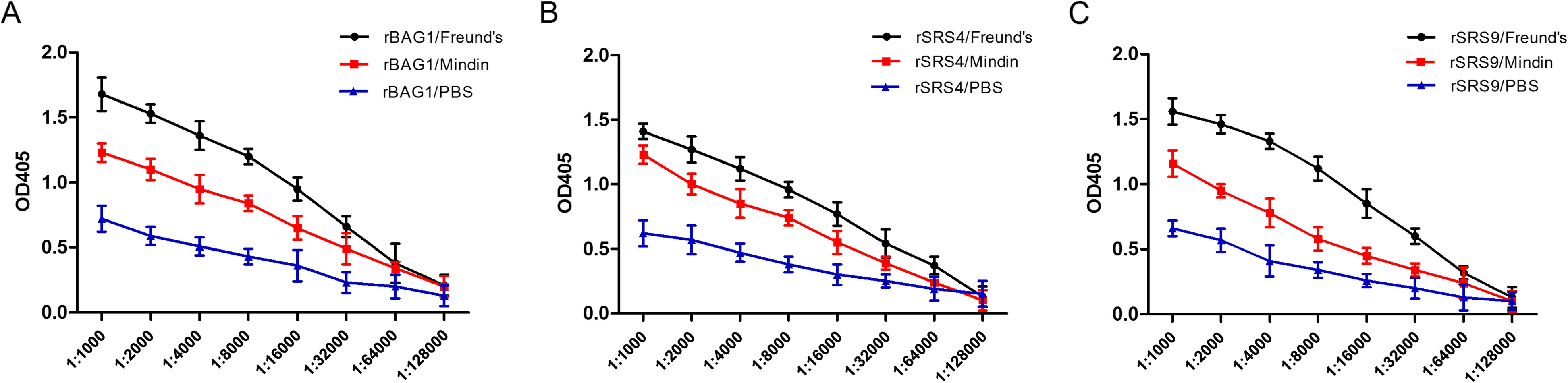

Supplement: Supplementary file 2 — Authors’ original file for figure 2 [file 12879_2014_3729_MOESM2_ESM.tif]

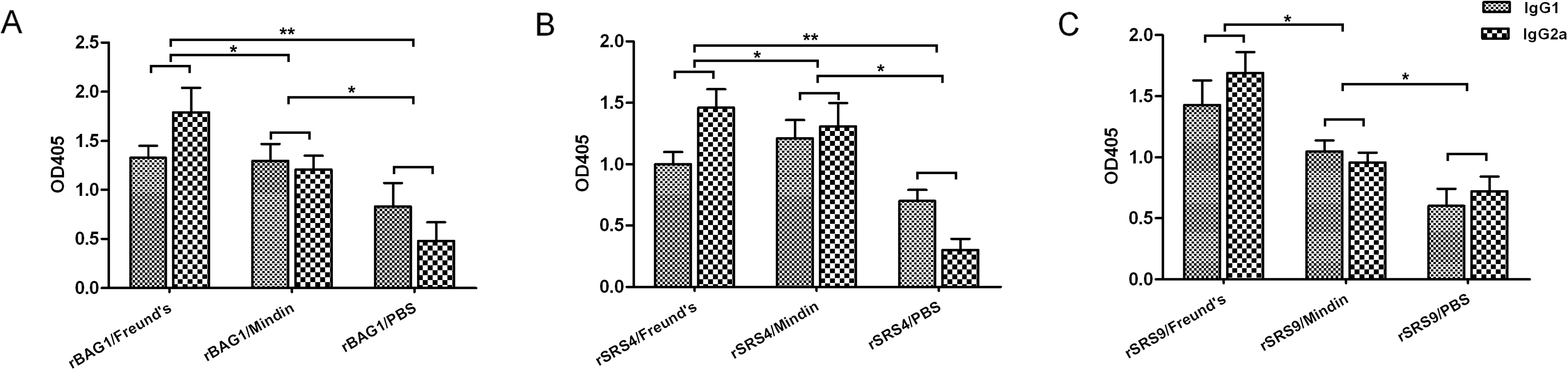

Supplement: Supplementary file 3 — Authors’ original file for figure 3 [file 12879_2014_3729_MOESM3_ESM.tif]

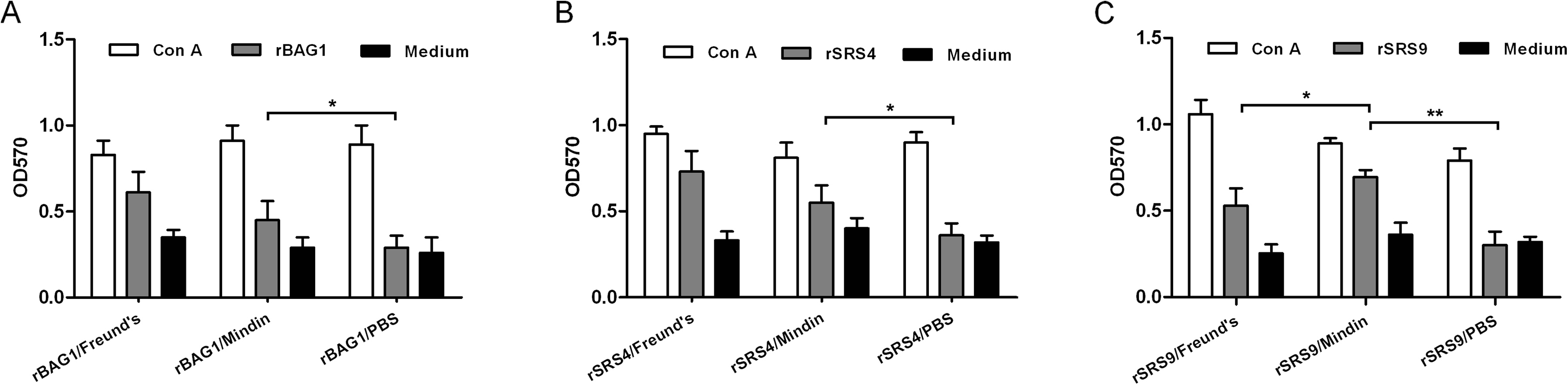

Supplement: Supplementary file 4 — Authors’ original file for figure 4 [file 12879_2014_3729_MOESM4_ESM.tif]

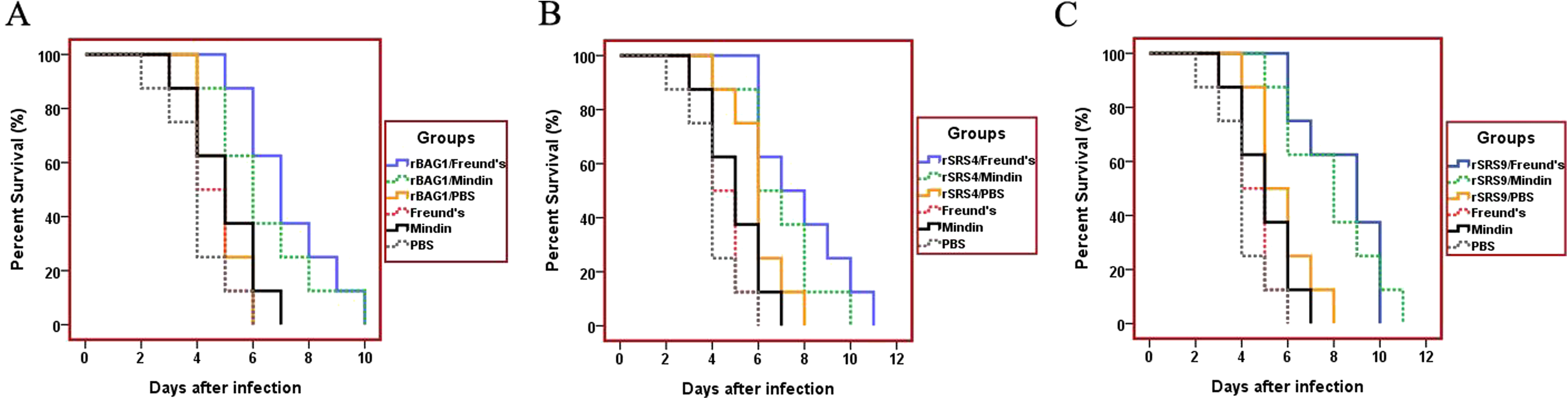

Supplement: Supplementary file 5 — Authors’ original file for figure 5 [file 12879_2014_3729_MOESM5_ESM.tif]
